# Epigenetic inactivation of inhibitor of differentiation 4 (Id4) correlates with prostate cancer

**DOI:** 10.1002/cam4.16

**Published:** 2012-08-02

**Authors:** Pankaj Sharma, Swathi Chinaranagari, Divya Patel, Jason Carey, Jaideep Chaudhary

**Affiliations:** Center For Cancer Research and Therapeutics Development, Clark Atlanta UniversityAtlanta, Georgia

**Keywords:** Epigenetics, Id4, prostate cancer

## Abstract

The inhibitor of DNA-binding (Id) proteins, Id1–4 are negative regulators of basic helix-loop-helix (bHLH) transcription factors. As key regulators of cell cycle and differentiation, expression of Id proteins are increasingly observed in many cancers and associated with aggressiveness of the disease. Of all the four Id proteins, the expression of Id1, Id2, and to a lesser extent, Id3 in prostate cancer and the underlying molecular mechanism is relatively well known. On the contrary, our previous results demonstrated that Id4 acts as a potential tumor suppressor in prostate cancer. In the present study, we extend these observations and demonstrate that Id4 is down-regulated in prostate cancer due to promoter hypermethylation. We used prostate cancer tissue microarrays to investigate Id4 expression. Methylation specific PCR on bisulfite treated DNA was used to determine methylation status of Id4 promoter in laser capture micro-dissected normal, stroma and prostate cancer regions. High Id4 expression was observed in the normal prostate epithelial cells. In prostate cancer, a stage-dependent decrease in Id4 expression was observed with majority of high grade cancers showing no Id4 expression. Furthermore, Id4 expression progressively decreased in prostate cancer cell line LNCaP and with no expression in androgen-insensitive LNCaP-C81 cell line. Conversely, Id4 promoter hypermethylation increased in LNCaP-C81 cells suggesting epigenetic silencing. In prostate cancer samples, loss of Id4 expression was also associated with promoter hypermethylation. Our results demonstrate loss of Id4 expression in prostate cancer due to promoter hypermethylation. The data strongly support the role of Id4 as a tumor suppressor.

## Background

The inhibitor of DNA-binding (Id) proteins, Id1–4 are negative regulators of basic helix-loop-helix (bHLH) transcription factors. The bHLH transcription factors regulate tissue-specific transcription and regulate many developmental pathways [1]. Structurally, the core HLH domain between Id and bHLH proteins is highly conserved that allows efficient Id-bHLH dimerization. However, the Id-bHLH dimer is transcriptionally inactive due to the lack of DNA-binding basic domain in Id proteins [2–4]. The interference of Id proteins with the key regulatory bHLH proteins is therefore an important interaction for proliferation and differentiation. The repertoire of Id-regulated cellular pathways is large and diverse due to their ability to interact and modulate the activity of bHLH and non-bHLH transcription factors and regulatory molecules (reviewed in [5–12]). As key regulators of cell cycle and differentiation, the expression of Id proteins is increasingly observed in many cancers and in most cases associated with aggressiveness of the disease including poor prognosis [13–16], metastasis [17], and angiogenesis [18, 19]. Of all the four Id proteins, the expression of Id1, Id2, and to a lesser extent, Id3 in cancer and the underlying molecular mechanism is relatively well known. On the contrary, epigenetic silencing of Id4 in many cancers tends to support its role as a tumor suppressor [20].

Paradoxically, Id4 appears to demonstrate both pro-tumor and anti-tumor properties. Epigenetic silencing of Id4 in leukemia [21], breast [22, 23], colorectal [24] mouse and human chronic lymphocytic leukemia (CLL [25]), and gastric cancer [26] tend to support its anti-tumor activity. Whereas high Id4 expression in a B-cell acute lymphoblastic leukemia [27] and B-cell precursor acute lymphoblastic leukemia(BCP-ALL [28]) due to *t*(6;14) (p22;q32) chromosomal translocation and in bladder [29] and rat mammary gland carcinomas [30] suggests that it may have pro-tumor activity also.

Based on data mining of published microarray databases in Oncomine database, we have shown that Id4 is highly expressed in the normal, normal adjacent, and benign prostates and its expression is significantly decreased in prostate cancer (metastatic prostate cancer <prostate cancer, data summarized in [20]). However, these observations are contradictory to an earlier study that demonstrated increased expression of Id4 in prostate cancer but negligible expression in the normal prostate [31]. Our previous studies also suggested that Id4 is regulated by androgens in normal prostate epithelial cells [32] and in androgen-sensitive prostate cancer cell line LNCaP [33]. Id4 expression is low in PC3 prostate cancer cells but undetectable or weakly expressed in androgen-independent DU145 prostate cancer cells due to promoter hypermethylation [20]. Ectopic Id4 expression also attenuates cell proliferation in DU145 cells that is associated with increased expression of cyclin-dependent kinase inhibitors p21and p27 [20]. Collectively, the data from our laboratory [20, 32, 33] demonstrated that Id4 acts as a potential tumor suppressor but its expression in prostate tissue is at best conflicting. In this study, we expand our observations of Id4 expression in established prostate cancer cell lines and prostate cancer tissue to demonstrate that Id4 expression is decreased in prostate cancer due to promoter hypermethylation. These results together with our previous mechanistic studies [20] strongly support the role of Id4 as a tumor suppressor in prostate cancer.

## Methods

### Cell lines and cell culture

Human prostate cancer cell lines PC3, DU145, and LNCaP were obtained from American Type Culture Collection (ATCC, Rockville, MD). C-33 and C-81 cells were kindly provided by Prof. Ming-Fong Lin (Department of Biochemistry and Molecular Biology and Eppley Institute for Cancer, University of Nebraska Medical Center, Omaha, NE) [34]. PC3 and DU145 cells were cultured in Ham's F12 (Gibco, Carlsbad, CA) medium containing 5% Bovine Calf Serum (Hyclone, Logan, UT) with appropriate antibiotics (pen/strep, fungizone, and gentamycin). LNCaP cells were cultured in RPMI with 5% fetal calf serum (FCS) and antibiotics. DU145 cells ectopically expressing human Id4 were prepared as reported previously [20]. Cells were cultured at 37°C in a fully humidified atmosphere containing 5% CO_2_.

### Prostate tissue samples

Formalin-fixed and paraffin-embedded 10 *μ*m sections in duplicate from age-matched prostate cancer (mean age 64.3 ± 2.4) and benign prostate hyperplasia (mean age 61.8 ± 3.1) affixed on Leica polyethylene naphthalate (PEN) membrane–coated slides were obtained from Cooperative Human Tissue Network (CHTN), Southern Division at University of Alabama at Birmingham and from Dr. Meenakshi Vij MD (Pathology), West Georgia Hospitals, LaGrange, GA, following appropriate IRB approvals. The Gleason score was available for each sample but the pre-operative PSA values were unavailable. The corresponding 5 *μ*m hematoxylin/eosin-stained tissue sections on glass slides were also obtained to assess and identify the cancerous regions for laser capture micro-dissection of tissue on Leica PEN slides. Before laser capture microdissection, the sections were briefly stained with hematoxylin and compared to the corresponding hematoxylin/eosin-stained section. The regions showing >75% cancerous regions or more than >80% normal/benign regions were dissected using Leica LMD6500 and captured in microcentrifuge tubes.

### DNA methylation analysis

Id4 promoter methylation was analyzed using methylation-specific PCR (MSP) as described previously [20, 23]. The MSP region amplified in context of the Id4 genome in this study has been previously investigated and well characterized in gastric [26], breast [22, 23], and colorectal cancers [24]. Briefly, genomic DNA from cell lines was isolated using DNeasy kit (Qiagen, Valencia, CA) and from laser-captured sections using ZR Genomic DNA tissue MicroPrep Kit (Zymo Research, Irvine, CA). Approximately 1 *μ*g of DNA was sodium bisulfite–modified using EZ DNA methylation Kit (Zymo Research) and subjected to MSP as described previously [20, 23]. The un-methylated specific primers (USP, U-reaction) that specifically hybridized with the un-methylated Id4 promoter sequence were forward 5′ (−194 to −166 bp) GGT AGT TGG ATT TTT TGT TTT TTA GTA TT-3′ and reverse 5′ (−60 to −33 bp) AAC TAT ATT TAT AAA ACC ATA CAC CCC A-3′ (reverse). The methylation specific primers (MSP, M – reaction) that specifically hybridized with methylated Id4 promoter sequence were forward 5′ (−192 to −166 bp)-TAG TCG GAT TTT TCG TTT TTT AGT ATC-3′ and reverse 5′ (−60 to −35 bp)-CTA TAT TTA TAA AAC CGT ACG CCC CG-3′. Polymerase chain reactions were performed in a 25 *μ*L reaction using GoTaq Green master mix (12.5 *μ*L, Promega) with 500 pm each of the 5′ and 3′ primers. Temperature conditions for PCR were as follows: 40 cycles of 94°C for 30 sec, 58°C for 45 sec, and 72°C for 30 sec, followed by 1 cycle at 72°C for 10 min. PCR products were separated on 1.5% agarose gels and visualized using GelDoc XR+ (BioRad, Hercules, CA).

### Immuno-histochemistry (IHC) of tissue microarray slides

Prostate cancer tissue microarrays were used to investigate Id4 expression in 54 prostate cancers (*n* = 7 for stage I, *n* = 22 for stage II, and *n* = 25 for stage III), 11 BPH, and 9 normal prostate core biopsies (1.5 mm) in duplicate (BC19014, BC19111, PRC481, and T192; BioMax, Inc., Rockville, MD). The cancer stage and histological type information for each core biopsy was available from the manufacturer for each of the sections. The mean age (mean ± SEM) of normal (normal + benign) and cancer samples were 66.9 ± 5.3 and 71.2 ± 4.9, respectively. The pre-operative PSA levels for cancer samples were not available.

Tissue microarray slides were de-paraffinized in xylene and re-hydrated through standard protocols. Antigens were retrieved by autoclaving in 0.01 M sodium citrate buffer pH 6.0 at 121C/20 psi for 30 min. The slides were then blocked for peroxidase activity in 3% H_2_O_2_ (in PBST: PBS with 0.05%Tween 20) for 10 min and then blocked in 10% goat serum (PBST with 1% BSA) for 2 h at room temperature. The blocked sections were incubated overnight at 4°C with primary antibody (1% BSA in PBST). The slides were then washed twice with PBST for 5 min each, and then incubated with secondary antibody (1% BSA in PBST, 1:1000, SA1-9510, HRP- goat anti-rabbit; Thermo Scientific, Rockford, IL) for 1 h. The slides were washed with PBST for 5 min and stained with DAB for 2 min. Slides were then finally counterstained in hematoxylin and mounted with Immuno-mount (Thermo Scientific), examined and photo-micrographs taken using the Zeiss fluorescent microscope with an AxoimCam version 4.5 imaging system.

### RNA preparation and RT-PCR

Total RNA was extracted using TRIzol (Invitrogen, Carlsbad, CA) as described previously [33]. The reverse transcribed [20] RNA was used to perform PCR using Id4 and *β*-actin specific primers [33]: Id4 (NM_001546): Forward (370 bp) 5′-ATG AAG GCG GTG AGC CCG GT-3′ and Reverse (843 bp) 5′-AAT GCT GTC GCC CTG CTT GTT; actin (NM_001101): forward (688 bp) 5′-GCG GGA AAT CGT GCG TGA CAT T and reverse (920 bp) 5′-GAT GGA GTT GAA GGT AGT TTC GTG.

### Western blot analysis

The prostate cancer cell lines were cultured on 75-mm plates in their respective media. Cells (5 × 10^6^) were washed once with ice-cold PBS and lysed in M-PER (Thermo-Scientific). Total cellular protein was prepared and Western blot analysis was performed using rabbit monoclonal anti-hId4 (BCH-9/82-12-50; Biocheck Inc., Foster City, CA) [33].

### GST-Id4 purification

Glutathione S-transferase (pReceiver-B04) fused in frame to protein coding region of human Id4 (GST-Id4) plasmid was custom synthesized by Genecopoeia. Plasmid was transformed into BL21 (DE3) competent cells (Novagen, Darmstadt, Germany). Protein expression in freshly grown cultures at 37°C was induced by 1 mM IPTG at 30°C. Four hours after induction, the BL21 (DE3) cells were centrifuged. The pellet was lysed at room temperature for 15 min in B-PER (Thermoscientific, Inc.) with DNase (3 Units) and lysozyme (100 *μ*g). The lysate was then centrifuged at 10,000 rpm for 10–15 min at 4°C. Recombinant GST-Id4 was affinity purified using GST fusion protein purification column (GeneScript) according to the manufacturer's protocol.

### Real-time quantitative PCR for analysis of Id4 expression on RNA purified from FFPE prostate samples

Unstained LCMD sections were obtained as above from prostate cancer regions that were either hypermethylated (*n* = 10), partially methylated (*n* = 7), and un-methylated benign or adjacent normal (*n* = 9) regions. The samples were used to purify RNA using Qiagen FFPE RNA isolation kit. The purified RNA was not quantifiable due to low volume and concentration. To circumvent this issue, 5ul of the purified RNA was reverse transcribed by reverse (3′) primer of Id4 or actin real-time primers (see below). The gene-specific reverse-transcribed RNA was then used to quantify Id4 and actin as described previously [20]. The ΔCt values (Id4-Ct subtracted from actin Ct) and ΔΔCt (fold change as compared to Id4 expression in benign samples) was used as a quantitative measure of Id4 expression. The real-time primers used for the quantitation of *β*-actin and Id4 were as follows: *β*-actin (amplicon length 140 bp: 1359–1498 bp) forward 5′-CTG GAA CGG TGA AGG TGA CA and reverse 5′-AAG GGA CTT CCT GTA ACA ATG CA; Id4 (amplicon length 127 bp: 560–687 bp) forward 5′-TGC AGT GCG ATA TGA ACG AC and reverse 5′-AG CTG CAG GTC CAG GAT GTA. The efficiency for actin and Id4 primers was 99.6 (slope –3.56) and 98.3 (slope –3.77), respectively.

### Statistical analysis

Student's *t*-test was used to calculate differences between paired observations as noted in figure legends. The Cohen's kappa coefficient was used as a measure of inter-observer reliability for assessing Id4 staining in TMA slides. Non-parametric Kruskal–Wallis one-way analysis of variance (ANOVA) for multiple comparisons followed by post hoc Dunn' multiple comparisons test was then used to infer statistical differences between Id4 staining in normal/benign and prostate cancer samples. Mann–Whitney *U*-test, Wilcoxon signed rank test and unpaired *t*-test with Welch's correction were used to compare methylation between normal (benign, ANP, and normal) and cancer ordinal data sets. For all analyses, a *P*-value less than 0.05 was considered significant. Statistical analyses were performed with either Graph Pad Prism (*t*-test, Mann–Whitney *U*-test, Wilcoxon signed rank test, and unpaired *t*-test with Welch's correction) or SPSS (Kruskal–Wallis and Kappa coefficient). All data are expressed as mean ± SEM.

## Results

### Id4 expression and methylation in prostate cancer cell lines

**Figure 1:**
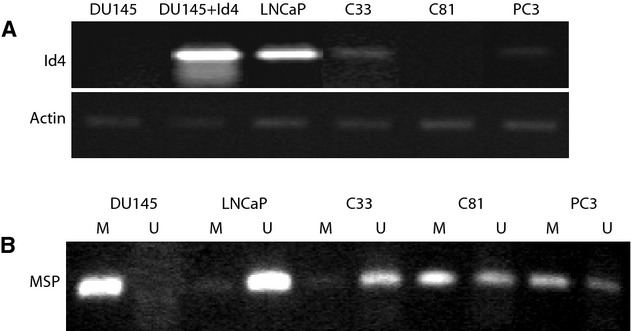
(A) Id4 expression in prostate cancer cell lines DU145, DU145 + Id4, LNCaP, C33, C81, and PC3. Id4 expression is not observed in DU145 and C81 cells. The androgen receptor–positive and androgen-sensitive LNCaP cells express Id4, however, C81 (derivatives of LNCaP) are androgen receptor positive but androgen insensitive, lack Id4 expression. DU145 cells stably transfected with Id4 expression plasmid (DU145 + Id4) were used as a positive control for Id4 expression. (B) Id4 promoter methylation in prostate cancer cell lines. M – methylated and U – un-methylated. A PCR band in the “M” lane represents promoter methylation. A PCR band in the “U” lane represents the un-methylated promoter. The band in both M and U lanes represents partial methylation. The Id4 expression in cell lines shown in panel A corresponds with the corresponding methylation pattern suggesting that Id4 is epigenetically regulated. Representative data from three separate experiments is shown.

Our previous studies have demonstrated that Id4 expression is high in LNCaP cells, low in PC3 cells and essentially absent in DU145 cells. Lack of Id4 expression in DU145 cells is due to promoter hypermethylation as shown in our previous study [20]. As LNCaP cells are less tumorigenic than DU145 and PC3 cells, we hypothesized that LNCaP derived cell lines, such as LNCaP-C33 and LNCaP-C81, which are significantly more tumorigenic may have less Id4 expression due to promoter hypermethylation. LNCaP, LNCaP-C33, and LNCaP-C81 recapitulate many characteristics associated with progression of prostate cancer cells from androgen-dependent to androgen-refractory phenotype [34]. Consistent with our hypothesis, negligible Id4 expression was observed in the androgen-independent and highly tumorigenic LNCaP-C81 cells (Fig. 1). The LNCaP-C33 cells retain partial androgen sensitivity and expressed Id4 that was significantly lower than parental LNCaP cells (Fig. 1). The Id4 expression in the cell lines correlated well with its promoter methylation: Id4 promoter was un-methylated in LNCaP cells and was partially methylated in LNCaP-C31 and LNCaP-C81 cells (Fig. 1). The DU145 cells were used as a positive control for associating Id4 expression and its promoter methylation [20]. These results demonstrated that Id4 expression is progressively lost in more aggressive prostate cancer cell lines due to promoter hypermethylation.

### Id4 expression in prostate cancer and normal prostate

**Figure 2:**
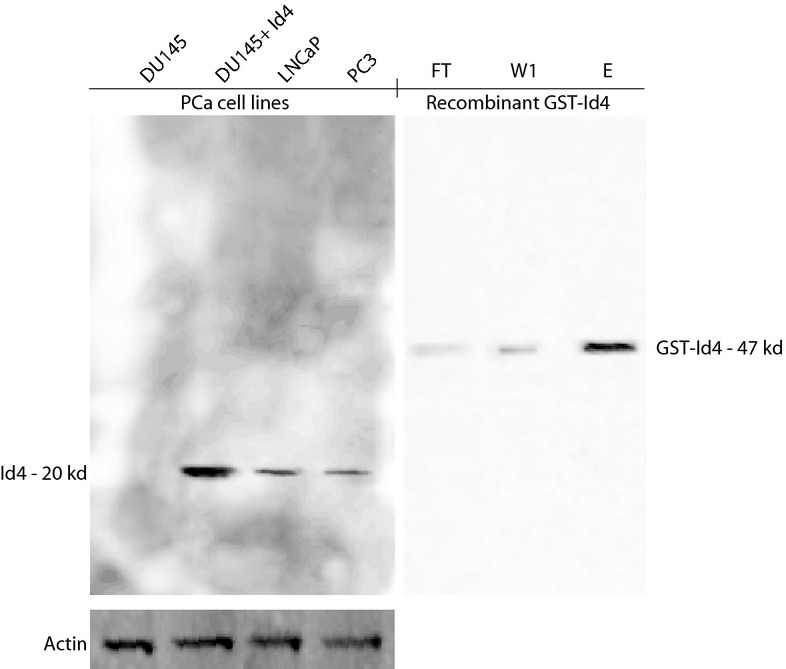
Id4 antibody characterization. The left panel is the Western blot analysis of Id4 expression in prostate cancer cell lines DU145, DU145 + Id4, LNCaP, and PC3. DU145 + Id4 cell line (see Fig. 1 legend for description) was used as a positive control for Id4 expression whereas parental DU145 cells were used as negative controls. Actin was used as a loading control. The Western blot is representative of three different analyses. The right panel shows the Id4 antibody specificity for recombinant GST-Id4. The lanes are as follows: FT, flow through from the GST affinity column; W1, first wash after flow through and; E, elute – elution of the bound GST-Id4 by glutathione. The Western blot was performed using 1 *μ*g of protein and is representative of three analyses.

We next investigated the expression of Id4 in prostate cancer tissue. A previous study reported increased Id4 expression with increasing grade of prostate cancer [31]. These results were inconsistent with Id4 expression in cell lines (discussed above), with our data mining [20] and other gene expression [35] studies that demonstrated decreased Id4 expression in prostate cancer. We therefore re-evaluated Id4 expression in prostate cancer tissue using a highly specific anti-human Id4 rabbit monoclonal antibody BCH-9/82-12-50. The BCH-9/82-12-50 antibody was monospecific for Id4 as demonstrated in Figure 2. A single Id4 reactive band was observed in LNCaP, PC3, and DU145 cells that were stably transfected with Id4 expression plasmid (DU145 + Id4). No Id4 protein expression was observed in DU145 cells in which Id4 promoter is methylated. These results were also consistent with Id4 mRNA expression (Fig. 1). The specificity of BCH-9/82-12-50 was further confirmed by using purified recombinant GST-Id4 protein that yielded a single specific band in Western blot analysis (Fig. 2).

**Figure 3:**
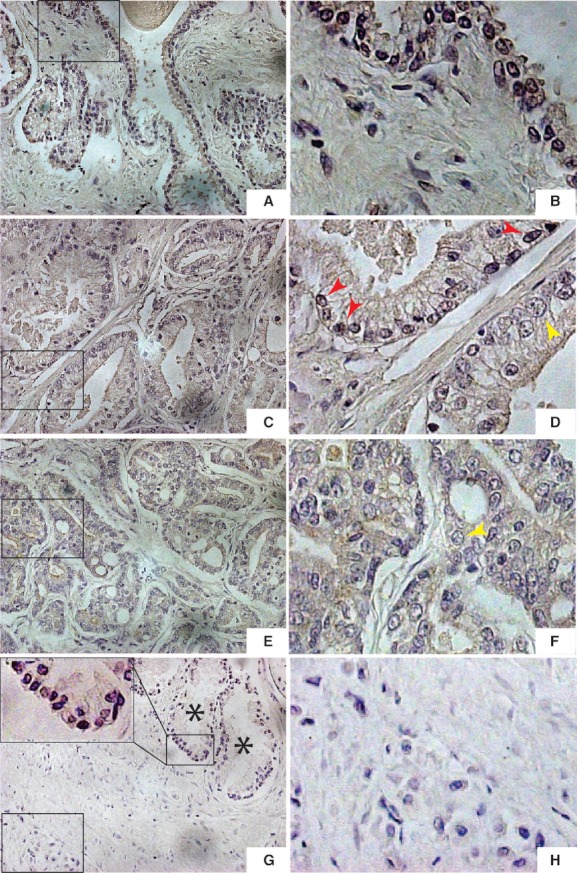
Prostate cancer tissue microarrays were used to investigate Id4 expression. Please refer to materials and methods for TMA details and sample size. Id4 was highly expressed in normal prostate (A) 200× and (B) 400× as seen by intense brown staining in the nuclei. Overall, Id4 expression decreased with increasing grade of prostate cancer (C) grade I (200×), (D) grade I (400×), (E) Grade II (200×), (F) grade II (400×), (G) Grade III (200×), and (H) Grade III (400×). The sections are also representative of scores used to quantify staining intensity: A and B – score 3; C and D – score 2; E – score 0. Id4 is mostly nuclear as seen by intense nuclear staining (brown, indicated by red arrow in D). At higher stages a clear large nucleus with no apparent brown staining is observed (yellow arrow in D and F). The sections were counterstained with hematoxylin that is reflected in the blue nuclei observed primarily in prostate cancer sections with undetectable Id4 expression. The 400× images in panels B, D, F, and H are corresponding images of boxed regions shown in panels A, C, E, and G (200×). The inset in panel G is the 400× image of the region showing high Id4 expression in normal prostate adjacent (asterisk) to cancer (stage III). Representative images are shown. The semi-quantitative analysis of all TMA sections analyzed for Id4 expression is shown in Figure 4.

Id4 immuno-histochemistry was performed on normal/benign prostate (*n* = 20, disease free) and prostate cancer (*n* = 54: stage I–III) tissue microarrays to determine their association with prostate cancer. Id4 expression was low to undetectable in majority of prostate adenocarcinoma (Fig. 3C–H, stage I–III) whereas 100% of the normal and benign prostate tissue (Fig. 3A and B: 200× and 400×, respectively) showed strong Id4 expression. Id4 expression was primarily nuclear and was occasionally observed in stage I (Fig. 3C and D, red arrows) but rarely observed in stage II and III prostate cancers (Fig. 3E–H). Interestingly, Id4 staining was also observed in seemingly normal tubules (Fig. 1G and H, indicated by asterisk) adjacent to cancer. These results further support the observations that decreased Id4 expression is a specific cancer-associated event.

**Figure 4:**
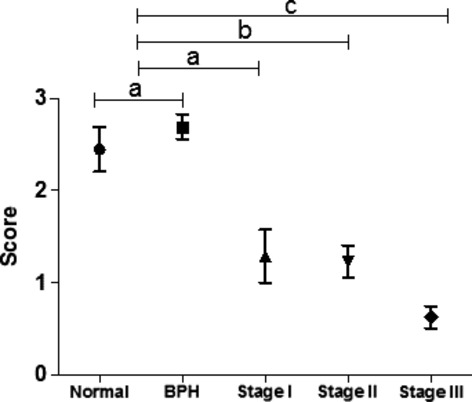
Semi-quantitative analysis of Id4 expression in normal prostate, BPH and prostate cancer (expressed as mean ± SEM). The intensity of staining was rated from 0 for below the level of detection (e.g., Fig. 3G and H) to 3 for strongest expression (e.g., Fig. 3A and B). The kappa correlation coefficient between the assessment of Id staining by two independent observers was 0.89 (linear weighting) and 0.94 (quadratic weighting). The statistical significance was calculated with non-parametric Kruskal–Wallis statistics followed by post hoc Duns's test for paired comparisons. The analysis demonstrates that Id4 expression is negatively correlated with progression of prostate cancer. The data are expressed as mean ± SEM of scores given by each observer. Please refer to Materials and Methods and Results section for sample size and statistical analysis. (A) Non-significant, (B) *P* = 0. 0.0023**, and (C) *P* > 0.0001***.

The intensity of staining was rated from 0 for below the level of detection to 3 for strongest expression (e.g., Fig. 3A was scored as 3, 3D was scored as 2, 3E was scored as 0) by two independent observers. The Cohen's kappa correlation coefficient between the assessment of Id4 staining by these two independent observers was 0.89 (linear weighting) and 0.94 (quadratic weighting). Non-parametric Kruskal–Wallis analysis followed by post hoc Dunn' multiple comparisons test was used to determine statistical differences between Id4 staining intensity in normal prostate and prostate cancer tissue microarray specimens (Fig. 3). The chi square of 16.21 was less than Kruskal–Wallis statistic H = 43.05 at *P* < 0.0001 providing strong evidence of significant differences between groups (Fig. 4). The post hoc Dunn's test suggested a significant difference between the intensity of Id4 staining between normal and stage II (*P* = 0.0023) and between normal and stage III (*P* < 0.0001). Unpaired *t*-test with Welch's comparison had the following *P*-values: normal versus BPH *P* = 0.387 (non-significant), BPH versus stage I *P* = 0.0021 (significant), BPH versus stage II *P* < 0.0001 (significant), and BPH versus stage III *P* < 0.0001 (significant) (Fig. 4).

### Id4 promoter is hypermethylated in prostate cancer

A strong correlation between Id4 expression and its promoter hypermethylation in prostate cancer cell lines was observed (Fig. 1). These results raised the possibility that the lack of Id4 expression in prostate cancer (Fig. 3) could be due to promoter hypermethylation. Laser capture micro-dissection (LCMD) was used to examine Id4 methylation in 41 prostate cancer samples, 19 benign and adjacent normal regions and 4 benign stroma adjacent to prostate cancer regions. The available Gleason grade with corresponding methylation (M)/un-methylation (U) status is summarized in Table 1. A PCR product using MSP was observed in 34/41 (83%, ranked as 3 for statistical analysis, see below) prostate cancer samples dissected by LCMD confirming Id4 methylation (Table 1). Occasionally (seven samples, 17% Table 1), a PCR product was also observed in the un-methylated PCR reaction suggesting that Id4 promoter is partially un-methylated (ranked as 2 for statistical analysis) in prostate cancer specimens. In contrast, Id4 promoter was un-methylated in 13 of 19 (69%, ranked as 1 for statistical analysis) benign or benign adjacent regions. Complete promoter hypermethylation was observed in only one benign sample (5%) whereas partial methylation was observed in 5/19 (26%) benign or benign adjacent regions. Id4 promoter hypermethylation was also present in 3/4 (75%) benign stromal samples, as expected, that is consistent with the lack of Id4 expression in stroma (Table 1 and Fig. 3). Comparison between benign (*n* = 19) and cancer (*n* = 41) samples by the paired Mann–Whitney test, Wilcoxon signed rank test, and unpaired *t*-test with Welch's correction revealed significant statistical differences (*P* < 0.001 and Mann–Whitney *U*-value of 41.5, *P* = 0.0004 in Wilcoxon rank test, and *P* < 0.0001 in unpaired *t*-test with Welch's correction). Due to small sample set the benign stromal samples (*n* = 4) were not included in the statistical analysis.

**Table 1:** Status of Id4 methylation in prostate cancer

No.	Diagnosis	Gleason grade/stage^*^	Methylation status	No.	Diagnosis	Gleason grade/stage*	Methylation status
1	Benign		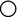	33	Malignant	9	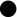
2	Benign		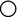	34	Malignant	9	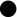
3	Benign		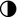	35	Malignant	8	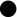
4	Benign		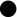	36	Malignant	9	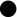
5	Benign		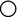	37	Malignant	6	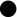
6	Benign		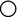	38	Malignant	8	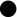
7	Benign		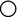	39	Malignant	7	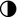
8	Benign		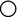	40	Malignant	7	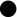
9	Benign		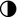	41	Malignant	6	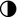
10	Benign (ANP)	8	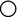	42	Malignant	8	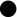
11	Benign (ANP)	7	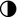	43	Malignant	8	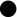
12	Benign (ANP)	8	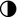	44	Malignant	9	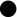
13	Benign (ANP)	8	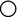	45	Malignant	8	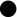
14	Benign (ANP)	6	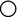	46	Malignant	7	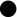
15	Benign (ANP)	7	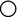	47	Malignant	7	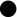
16	Benign (ANP)	7	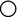	48	Malignant	7	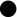
17	Benign (ANP)	6	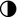	49	Malignant	7	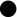
18	Benign (ANP)	8	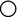	50	Malignant	7	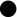
19	Benign (ANP)	5	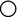	51	Malignant	7	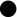
20	Benign stroma	7	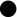	52	Malignant	6	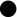
21	Benign stroma	8	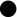	53	Malignant	7	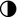
22	Benign stroma	8	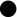	54	Malignant	7	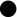
23	Benign stroma	8	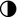	55	Malignant	7	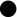
24	Malignant	2*	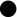	56	Malignant	8	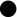
25	Malignant	2*	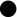	57	Malignant	7	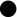
26	Malignant	3*	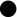	58	Malignant	8	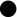
27	Malignant	3*	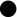	59	Malignant	9	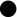
28	Malignant	3*	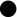	60	Malignant	7	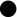
29	Malignant	2*	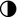	61	Malignant	7	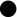
30	Malignant	8	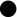	62	Malignant	7	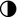
31	Malignant	8	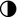	63	Malignant	NA	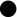
32	Malignant	7	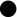	64	Malignant	NA	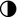

*, stage; 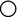, un-methylated; 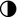, partially methylated; 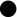, hypermethylated; ANP, adjacent normal prostate.

### Id4 promoter hypermethylation is associated with decreased Id4 expression in prostate cancer

**Figure 5:**
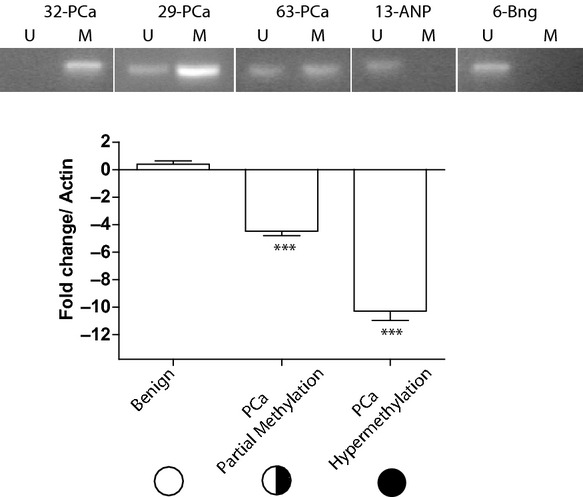
Id4 methylation is associated with its expression in prostate cancer. The top panel represents the MSP on selected prostate cancer and adjacent benign (ANP) and benign (Bng) samples. (The numbers corresponds to the sample numbers shown in Table 1.) A similar MSP was performed on all samples and the results are indicated in table 1. The bottom panel shows the quantitative real-time PCR of Id4 transcript in benign, prostate cancer samples with partial and complete methylation (benign *n* = 9; Pca partial methylation, *n* = 7 (all partially methylated samples in prostate cancer) and Pca hypermethylation *n* = 10, representative shown in top panel). The real-time data are represented as ΔΔCt, expressed as fold change as compared to actin and shown as mean ± SEM on Log2 scale (*y*-axis). The data are normalized to Id4 expression in benign samples set to 1. Actin was used to normalize the data (***P < 0.001, *t*-test).

A direct relationship between Id4 promoter methylation with Id4 expression by qRT-PCR was investigated in a subset of prostate cancer (*n* = 10 each for completely methylated and *n* = 7 for partially methylated prostate cancer samples) and benign prostate samples (*n* = 9). As shown in Figure 5, the Id4 expression by quantitative gene specific reverse transcriptase polymerase reaction on RNA purified from LCMD samples correlated with the corresponding Id4 promoter hypermethylation. High Id4 expression was observed in normal samples (normalized to 1 in ΔΔCt calculation) showing no Id4 promoter methylation. In prostate cancer samples, Id4 expression was clearly dependent on Id4 promoter hypermethylation: Id4 expression significantly decreased by 76 and 222-fold (essentially un-detectable) in partially methylated and completely methylated prostate cancer samples, respectively (Fig. 5). These analyses confirmed that Id4 promoter hypermethylation in prostate cancer results in decreased Id4 expression.

## Discussion

In this report, we demonstrate that Id4 expression is attenuated in prostate cancer due to promoter hypermethylation. This study strengthens our previous report which provided direct evidence that Id4 acts as a tumor suppressor in prostate cancer. The tumor suppressor role of Id4 appears to be unique as compared to other members of the Id gene family (Id1, Id2, and Id3) that may act as oncogenes or co-operating oncogenes in many cancers [6, 12, 36].

A recent report suggested a positive association between Id4 expression and prostate cancer metastasis [31]. On the contrary, we provide multiple lines of evidence that demonstrate decreased Id4 expression in prostate cancer. First, in LNCaP cell line-based prostate cancer progression model Id4 transcript is decreased from androgen-dependent LNCaP cells to androgen-independent LNCaP-C81 cells, with an intermediate expression observed in LNCaP-C-33 cells. Second, Id4 protein expression is significantly decreased and in most cases undetectable in advanced stages of prostate cancer as detected by a highly specific rabbit monoclonal antibody. Moreover, microarray studies ([35] and summarized in [20]) on clinically well-defined prostate cancer samples and analysis of a sub-set of clinical samples in this study also demonstrated decreased Id4 expression at the transcript level (mRNA). Thus, decreased Id4 expression in prostate cancer is observed at both transcript and protein level. At the mechanistic level, the transcriptional inactivation of Id4 is associated with aberrant promoter methylation in prostate cancer cell lines and tissue samples as demonstrated in this study and confirmed by others [37]. Our results are therefore consistent with the epigenetic silencing of Id4 due to promoter hypermethylation in cancers: T-/natural killer acute lymphoblastic leukemia [21], gastric [26], breast [23] colorectal [24], and prostate cancer [37].

The silencing of Id4 in cancers raises an important question: what is the normal physiological function of Id4 in at least those tissues which upon transformation leads to its loss of expression such as the prostate? Our earlier study [20] provided some answers at the mechanistic level: (1) Androgens up-regulate Id4 expression in normal prostate epithelial cell (PrEC) and (2) ectopic Id4 expression in androgen receptor negative DU145 cells leads to increased E-cadherin expression and decreased cell proliferation due to an S-phase arrest, increased expression of cyclin-dependent kinase inhibitors p21 and p27 and most importantly restoration of androgen receptor expression. The increase in the transcript of p27, p21, E-cadherin, and androgen receptor in DU145 cells suggests that Id4 over-expression modifies intracellular transcriptional pathways possibly through complex protein–protein interactions leading to restoration of transcriptional networks that are in general tumor-suppressive. Induction of Id4 by androgens in normal cells and restoration of androgen receptor in DU145 cells also suggests a potential feedback loop between AR and Id4. Perhaps, one of the mechanism by which AR becomes oncogenic could be due to its inability to trans-activate tumor suppressors such as Id4 due to promoter hypermethylation.

The HLH domain between Id4 and its other family members (Id1, I2, and Id3) is highly conserved thus supporting its role as a negative regulator of bHLH transcription factors [38]. The tumor-promoting properties of Id1, Id2, and Id3 are at least partially shared by Id4 also: Id4 has been shown to promote neoplastic transformation/growth. Increased Id4 expression is observed in acute lymphoblastic leukemia due to a t(6;14)(p22;q32) translocation [27]. Id4 expression is also associated with proliferation and invasiveness [30] in rat mammary gland carcinoma. Moreover, in breast cancer cells, Id4 and the tumor suppressor BRCA1 exist in a negative feedback loop [39–41]. But studies have also demonstrated epigenetic silencing of Id4 in breast cancer [22, 23]. Thus, even in cancers arising from the same organ such as the breast, Id4 may act as both tumor suppressor and tumor promoter [22, 23, 30, 39–41]. Evidence suggests that Id4 may share some functions with its family members but emerging data support the role of Id4 as a tumor-suppressive. We speculate that Id4 may have unique bHLH or non-bHLH interaction partners that could largely define its tumor-promoting versus tumor-suppressive functions. Support for this mechanism is based on the evidence that interactions of Id2 with Rb [42, 43] and polycystins [44], Id1 and Id3 with Ets [45] transcription factors largely contribute to their oncogenic potential by releasing cell cycle blockade at multiple levels [46]. Although all these mechanisms are largely tumor-promoting, similar tumor-suppressive interactions that are unique to Id4 could exist that remains to be investigated.

## Conclusions

Our results demonstrate that Id4 expression is decreased in prostate cancer due to promoter hypermethylation. Our results, in general agree with the majority of results that support the role of Id4 as a tumor suppressor due to epigenetic inactivation in other cancers. Contrary to these observations, studies have also demonstrated pro-tumor function of Id4 that is consistent with its other family members Id1, Id2, and Id3. In this regard, studies from breast cancer are particularly interesting that demonstrate both pro- and anti-tumor function of Id4. We speculate that these opposing roles of Id4 sometimes in the cancers originating from the same tissue could be due to specific Id4 interactions that are pro- or anti-tumor.
